# Disentangling Within- and Between-Person Effects During Response Inhibition in Obsessive-Compulsive Disorder

**DOI:** 10.3389/fpsyt.2021.519727

**Published:** 2021-03-25

**Authors:** Pernille Hagland, Anders Lillevik Thorsen, Olga Therese Ousdal, Rolf Gjestad, Stella J. de Wit, Bjarne Hansen, Kristen Hagen, Gerd Kvale, Odile A. van den Heuvel

**Affiliations:** ^1^Bergen Center for Brain Plasticity, Haukeland University Hospital, Bergen, Norway; ^2^Department of Clinical Psychology, University of Bergen, Bergen, Norway; ^3^Department of Radiology, Haukeland University Hospital, Bergen, Norway; ^4^Center for Crisis Psychology, University of Bergen, Bergen, Norway; ^5^Division of Psychiatry, Haukeland University Hospital, Bergen, Norway; ^6^Centre for Research and Education in Forensic Psychiatry, Haukeland University Hospital, Bergen, Norway; ^7^Department of Anatomy and Neurosciences, Amsterdam UMC, Vrije Universiteit Amsterdam, Amsterdam, Netherlands; ^8^Department of Psychiatry, Amsterdam Neuroscience, Amsterdam, Netherlands; ^9^Psychiatric Department, Hospital of Molde, Molde, Norway

**Keywords:** obsessive-compulsive disorder, response inhibition, stop signal task, functional magnetic resonance imaging, Bergen 4-day treatment, multilevel modeling

## Abstract

**Background:** Obsessive-compulsive disorder (OCD) has been related to worse performance, abnormal brain activity, and functional connectivity during response inhibition. Whether these findings are indications of stable traits that contribute to the development of the disorder, or whether they are a result of the state severity of obsessions and anxiety, remains unclear since previous research mainly has employed cross-sectional designs. The present study aimed to assess longitudinal between- and within-person relationships between symptoms, task performance, right inferior frontal gyrus brain activation, and connectivity between the right amygdala and the right pre-supplementary motor area in 29 OCD patients before and after concentrated exposure and response prevention treatment.

**Method:** Patients received exposure and response prevention delivered during 4 consecutive days, following the Bergen 4-day Treatment format. Patients performed a Stop Signal Task during 3T functional Magnetic Resonance Imaging the day before treatment, as well as 1 week and 3 months after treatment completion. Multilevel models were used to analyze disaggregated within- and between-person effects over time. Independent variables were scores on the symptom severity scales for OCD, anxiety, depression, and state distress during scanning. Dependent variables were reaction time for go trials, stop signal response time, task-related brain activation and connectivity.

**Results:** A positive between-person effect was found for obsessive-compulsive, anxiety, and depressive symptom severity on go trial reaction time, indicating that patients with higher symptom scores on average respond slower during accurate go trials. We also found no significant between- or within-person relations between symptom severity and task-related activation or fronto-limbic connectivity.

**Conclusions:** The between-person findings may point toward a general association between slower processing speed and symptom severity in OCD. Longitudinal studies should disaggregate between- and within-person effects to better understand variation over time.

Obsessive-compulsive disorder (OCD) is characterized by intrusive and persistent thoughts, images or impulses (obsessions) and time-consuming repetitive behaviors or rituals (compulsions) (1). The disorder affects approximately 1-3% of the population worldwide (2), and is often highly impairing (3). The majority of patients are affected before their mid-twenties, and if untreated, the disorder is often chronic (4). Patients' poor control over obsessions and compulsions has been related to problems in inhibitory control, which has been studied through interference and response inhibition tasks (e.g., Go-no go task, Stop Signal Task (SST), respectively) (5, 6).

Response inhibition is defined as “suppressing or resisting a prepotent (automatic) response to make a less automatic but task-relevant response” (7). In the SST, it refers to canceling an already initiated motor response, when a stop signal occurs (8). Deficits in response inhibition have been proposed as a possible endophenotype for OCD as longer response times on inhibitory tasks have been found in both OCD patients (8–10) and their unaffected first-degree relatives compared to controls (9, 10). Abnormal inhibitory processes in OCD patients compared to healthy controls have also been found in a meta-analysis of executive functioning in OCD patients (7). Whether this reflects characteristics of having OCD regardless of symptom level, or depends on the severity of disorder and thus will change after successful treatment, is not known.

Neuroimaging studies of response inhibition have shown functional activation in a fronto-parietal network, including the pre-supplementary motor area (pre-SMA), inferior frontal gyrus (IFG), caudate nucleus, thalamus, and subthalamic nucleus (11–14). De Wit et al. (8) found altered task-related blood-oxygen level dependent (BOLD) response during SST performance in the pre-SMA in both OCD patients and their unaffected siblings compared to healthy controls. Moreover, patients showed less task-related activation in the right IFG and inferior parietal cortex compared to healthy controls and siblings. In a recent meta-analysis of functional Magnetic Resonance Imaging (fMRI) studies on inhibition in OCD, Norman et al. (15) found that patients showed increased inhibition related activity in the parietal, temporal, and premotor cortices. They also found less activation in patients than controls during successful inhibition in striatum, anterior cingulate, frontal, and parietal cortices. The inconsistent findings between studies of inhibition in OCD could partly be explained by different studies using different response inhibition tasks, which might measure different aspects of response inhibition (5, 16). The Stop Signal and the Go/No go tasks assess motor inhibition, while tasks such as the Stroop Color Word Test examine cognitive interference (5, 17). The motor inhibition tasks also differ; the SST probes action cancellation, while the Go/No go assesses action suppression (18). The SST has more often than the Go/No go task shown behavioral differences between OCD patients and healthy controls, which may indicate that the SST taps more into the core pathology of OCD, namely to cancel an already initiated action (5, 16).

Whether altered brain activity and task performance in OCD are stable traits that contribute to the development of the disorder, or whether they are a result of obsessions and anxiety (i.e., state), remains unclear. A contemporary mechanistic model, the “executive overload model” (19), suggests that worse neuropsychological performance could be related to trait, limbic interference (state), or a combination of both, and some neuroimaging studies offer support for this model (20–23). This can be studied by investigating response inhibition before and after clinical change using longitudinal assessments in treatment studies.

OCD can be effectively treated using cognitive behavioral therapy (CBT), including exposure-*in vivo* and response prevention (ERP), and selective serotonin reuptake inhibitors (SSRI) (24). Some treatment studies using CBT reported effects of treatment on task performance (25–29), while others did not (30–32). Some studies reported increased task-related BOLD signal after treatment in the prefrontal, temporal and parietal cortices and putamen (30), whereas others reported decreased task-related activation after treatment in the frontal, temporal and parietal lobes, and hippocampal gyrus (25), and decreased insula activation (26). One study found abnormalities in inhibition-related activation in the ACC, frontal cortices in pediatric OCD patients compared with controls, that normalized after treatment. A treatment effect was also found in the insula during high-conflict trials, but only for older patients (26). Electroencephalogram (EEG) studies of error-related negativity (ERN) found increased ERN amplitudes during the Flanker task in both pediatric (33) and adult (34, 35) patients and their unaffected siblings compared to healthy controls, and this difference remained after symptom improvement (33, 36). This is in line with the finding of de Wit et al. (8) of more pre-SMA activation in OCD patients and siblings as a possible endophenotype of OCD.

We recently investigated task performance, activation and functional connectivity in 24 OCD patients and 19 healthy controls who performed the SST during fMRI before, 1 week after, and 3 months after concentrated exposure treatment, the Bergen 4-Day Treatment format (B4DT) (37, 38). We found significantly less right IFG activation in OCD patients compared to healthy controls during successful response inhibition before treatment. Pre-treatment, OCD patients also showed more inhibition-related connectivity between the right amygdala and the right pre-SMA than healthy controls. There were no significant changes directly after treatment and 3 months later, nor significant correlations with baseline symptom severity or symptom improvement after treatment, suggesting that inhibition-related neural correlates are stable markers of OCD (37). Furthermore, an exploratory multilevel analysis showed that more right amygdala-right IFG connectivity was related to slower stop signal reaction time (SSRT).

The study of Thorsen et al. (37) focused on differences in performance, activation, and connectivity before and after treatment using repeated-measures ANOVAs, which revealed no significant changes in patients at the group level. However, standard correlation, regression or ANOVA models do not allow for separating variation between and within individuals, and these methods are also limited in other ways. First, calculating and correlating change scores is controversial since they are highly dependent on the reliability and variance of the measure (39, 40). Second, correlating change scores requires a rapidly increasing number of tests for multiple time points, independent and dependent variables. Third, patients with incomplete information must be excluded, even though the data might be missing in an unsystematic way (41). Fourth, change scores contain information on both within-person variation (how much a person varied from their own mean at each time point) and between-person variability (e.g., persons with a higher baseline score can have more change, since variables are constrained within a specific range).

Researchers in clinical psychology and neuroimaging have recently adopted longitudinal multilevel (mixed) models (MLM) that solve many of these issues (42–44). First, the number of tests is reduced by having one longitudinal model per independent variable, where independent variables can have varying values at each time point. Second, such models can use all available information as long as data can be assumed to be missing at random (41). Third, they can fit a variety of covariance structures, so that measurement error can be allowed to correlate over time (41). Lastly and most importantly for the present study, they also allow for separating between- and within-person effects over time (45). Between- and within-person effects can be disentangled by person-centering the independent variable into two regressors: (1) the between-person variable consists of the participant's mean score across time (e.g., mean symptom severity before treatment, after, and at follow-up), and (2) the within-person variable is how much the person varies from their own mean (e.g., how much their symptoms are increased or decreased relative to their own mean) at each time point. This allows for testing both whether higher mean symptom severity across time and whether variation within the person is related to the dependent variable (46).

In the present study we sought to disentangle between- and within-person effects using longitudinal multilevel models. This is a substantial extension of our previous report (37) and should be regarded as exploratory due to its methodological novelty. The first aim was to investigate whether between-person effects of symptom severity and state anxiety with task performance, IFG activation, and fronto-limbic connectivity during inhibition could explain our previous findings (37). Second, we wanted to investigate the relation intra-individual variation between these variables over time. Finding significant between-person effects would further strengthen the trait hypothesis, whereas significant within-person effects could suggest that task performance and its neural correlates can also covary with symptom severity at the individual level, which can be complementary to between-person effects. All analyses were longitudinal and included available information from all three time points, which may provide greater power than the traditional analyses in Thorsen et al. (37).

## Methods

### Participants

We assessed 29 OCD patients who underwent the B4DT, and performed a SST during fMRI 1 day before treatment, 3 days after (1 week after the initial scan session), and 3 months after treatment. Symptoms of OCD, anxiety, depression, and subjective distress were independent variables. Dependent variables were go trial and stop signal reaction time, task-related BOLD signal in the right IFG during successful inhibition, and task-related functional connectivity between the right pre-SMA and the right amygdala during successful inhibition. Thirty five patients with OCD were recruited after being admitted to the B4DT at the Haukeland university hospital OCD-team in Bergen, Norway. The mean pre-treatment Yale-Brown Obsessive Compulsive Scale (YBOCS) score was 26.83. Seven patients used psychotropic medication pre-treatment, and continued on a stable dosage throughout treatment and follow-up. See Table 1 for clinical and demographic variables. The study was approved by the local ethics board (REC South-East, 2015/936), and all participants gave written consent before participation in the study.

**Table 1:** Clinical and demographic variables.

**Characteristic**	**OCD patients (*N* = 29)**
Age (years) (Mean)	30.9 (9.2)
Sex (*n* females [%])	18 (62.1)
Years of education (Mean [SD])	14.8 (2.4)
Pre-treatment YBOCS score (Mean [SD])	26.83 (4.11)
Pre-treatment GAD-7 score (Mean [SD])	12.62 (5.19)
Pre-treatment PHQ-9 score (Mean [SD])	11.45 (6.13)
Psychotropic medication (*n*, [%])	7 (24.1)
Right-handed (*n* [%])	28 (96.5)

*GAD-7, Generalized Anxiety Disorder 7; OCD, obsessive-compulsive disorder; PHQ9, Patient Health Questionnaire-9*.

Longitudinal (at least 2 time points) behavioral and brain imaging data were available for 29 participants. Of the 35 participants originally included, two participants did not complete the task at baseline due to discomfort or reading difficulties. Three patients only participated in the pre-treatment session. Two of these were unable to complete imaging due to discomfort during imaging, and the third was not motivated for further participation after recovery. One patient participated in the pre-treatment and follow-up sessions, but was excluded from the behavioral analyses because of too many errors on the go trials during the follow-up session. Twenty four of the patients included in the longitudinal analyses had behavioral and brain imaging data for all three time points. Of the five included patients with data for only two time-points, one declined participation in the follow-up fMRI session, and two did not participate in the follow-up fMRI session because of pregnancy. The remaining two had data for all three time points, but each had one of their sessions excluded from the analyses due to excessive movement. All participants completed the B4DT.

### Assessment

fMRI was conducted the day before treatment started (Monday), 3 days after the end of treatment (the following Monday), and 3 months after treatment.

Symptom severity was measured with YBOCS (47), and changes in symptom severity were measured at 1 week, and at 3 months follow-up. The interview was performed *via* telephone at each time point by trained clinical psychologists who were not involved in the treatment. Depressive symptoms were assessed with the Patient Health Questionnaire-9 (PHQ-9) (48), and severity of general anxiety symptoms were measured with the Generalized Anxiety Disorder 7-item (GAD-7) scale (49). PHQ-9 and GAD-7 are self-report forms that the patients completed at all time points. A total Y-BOCS score lower than 13 was defined as clinical remission, whereas response was defined as at least 35% reduction on the Y-BOCS (50). Upon completing each MRI sequence, participants were asked to verbally report their subjective level of distress on a scale from 0–100, where 100 represents the highest stress level imaginable, using the Subjective Units of Distress Scale (SUDS).

### Stop Signal Task

The participants performed a visual SST (51) during fMRI, where they responded to an arrow by pressing a button held in either their right or left hand, corresponding to the direction of the arrow. The participants were instructed to press as fast and correctly as possible when they saw the arrow (“go trial”), but to refrain from pressing the button if a cross appeared over the arrow (“stop trial”). The time between the appearance of the arrow and the cross during stop trials was continuously adjusted to make sure that the participants had a success rate of approximately 50%. The task lasted approximately 15 min, and was performed during the last half-hour of an MRI session with a total duration of 1 h.

The mean response time of correct go trials (SucGoRT) and the stop signal response time (SSRT) were used as behavioral measures in the analyses. Participants with <40% correct go trials or failed stop trials outside of the 25–75% range were excluded from the analyses (52). The integration method was used to calculate the reaction time for stop trials to ensure a more reliable estimate of the SSRT (53).

### Image Acquisition and Processing

fMRI images were acquired using a 3T General Electric Discovery MR750 with an eight-channel head coil at Haukeland university hospital, Bergen, Norway. Structural T1-weighted images were acquired using a 256 × 256 matrix, 192 slices, isotropic voxel size 1 mm^3^, TE = 30 ms, TR = 7,000 ms, flip angle = 12°, FoV = 256 mm. Functional images were acquired in 430 T2-weighted BOLD volumes, using a 64 × 64 matrix, 34 slices (2.8 mm thickness, 0.2 mm interslice gap), isotropic voxel size 3 mm^3^, TR = 2100 ms, TE = 30 ms, flip angle = 80°, FoV = 22 mm with interleaved slice excitation.

As described in Thorsen. et al. (37), SPM12 was used for preprocessing of the functional data. The data were slice time corrected, realigned and motion corrected. Unified segmentation was used for normalization, and voxels were resliced to 3 mm^3^ and smoothed to 8 mm with a full width at half maximum kernel. Accurate go and stop trials, and failed stop trials were modeled as 0 s events, together with six motion parameters at the first level. Then, to remove low frequency noise, a high pass filter with 128 s cutoff was applied. We investigated the following regions of interest using 10 mm spheres: the bilateral anterior insula/IFG, pre-SMA, operculum, inferior parietal cortex, and midline posterior cingulate cortex for successful inhibition, based on the results from a recent meta-analysis of the SST (14). For the present study we extracted parameter estimates during successful stop > successful go trials from the IFG (MNI 42, 23, −13).

The generalized psychophysiological interaction toolbox [gPPI, (54)] was used to model task-related functional connectivity, with the right (MNI 23, 0, −16) amygdala as a spherical 5 mm seed region. The PPI models included time course of the seed region, the three task regressors, three PPI regressors, and six motion parameters. Then, group comparisons were performed by entering successful inhibition (SucStop > SucGo) contrasts for activation and connectivity into second-level models. For the present study connectivity estimates were extracted from the right pre-SMA (MNI 3, 23, 56) using 6 mm spheres around the peak group differences from Thorsen et al. (37), using MarsBar.

### Statistical Analyses

For the exploratory analyses, a linear mixed effects model in SPSS 24 was used. Time specific outcome levels were analyzed as a function of between- and within-person predictors. Chi-square likelihood ratio tests were used to determine the best fitting covariance structure, and whether to use fixed or random intercept and slopes. All models were first tested with a scaled identity covariance structure and fixed intercepts and slopes. Different covariance structures and random intercepts and slopes where then sequentially tested until the best fit was reached (see **Table 3** for information on intercepts and slopes used for each model). Maximum likelihood was used as estimation method. In line with Wang & Maxwell (55), person-mean-centering was considered the most appropriate disaggregation method of within-person and between-person effects. Since symptom scores were expected to change over time due to treatment, detrending was not applied (55).

**Figure 1:**
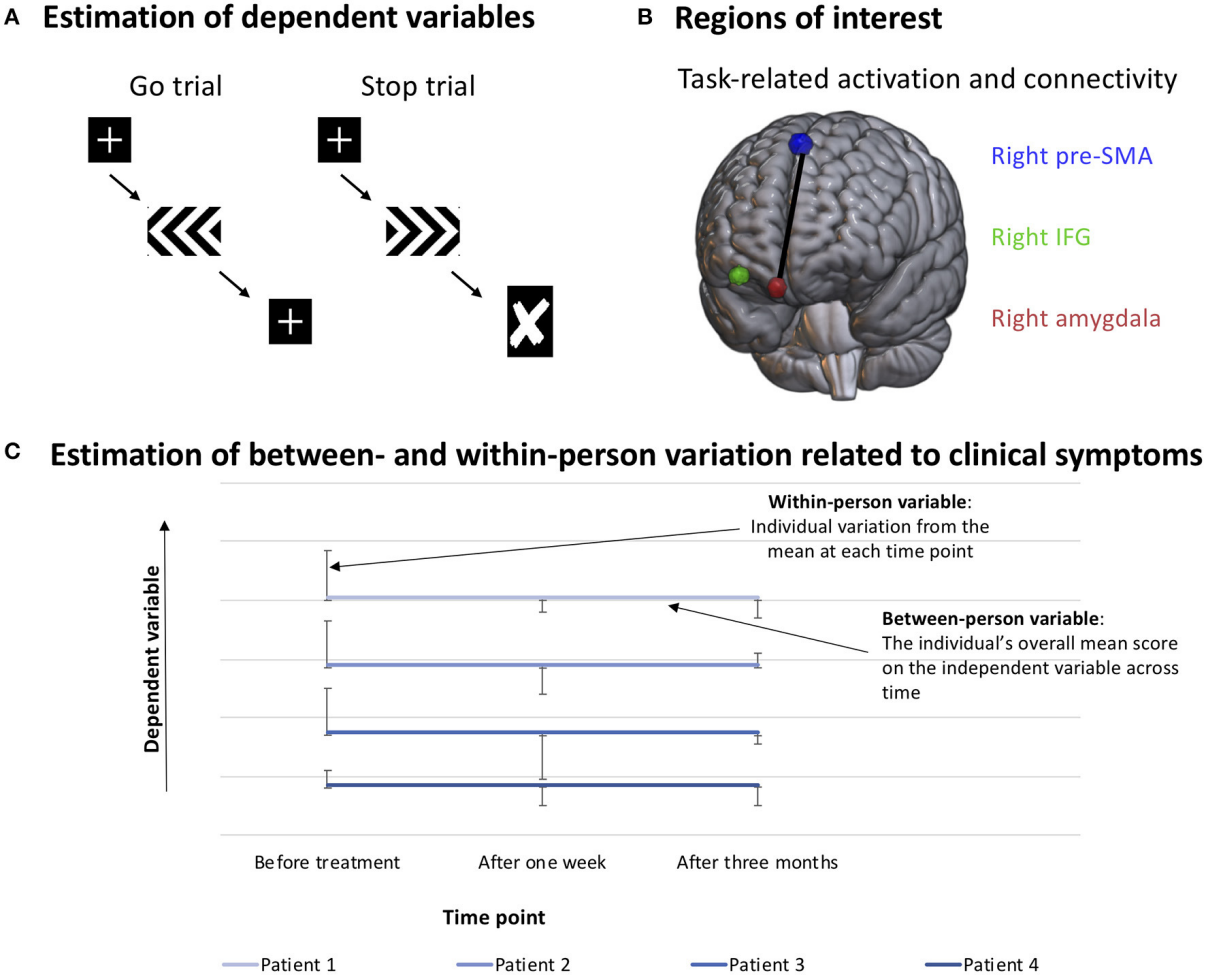
Method. **(A)** Reaction time during go trials (SucGoRT, left) and inferred reaction time on stop trials (SSRT, calculated using the integration method). **(B)** Visualization of regions of interest. Extracted beta values of task-related IFG activation and task-related connectivity between the right pre-SMA and the right amygdala were used as dependent variables. **(C)** Example of how the variables are organized when investigating between- and within-person effects. The between-person variable consists of the person's mean of their scores on each measurement time, while the within-person variable consists of the individual's deviation from their own mean score on each time point. This allows for the investigation of whether one or both of the variables predict variation in the dependent variable.

To examine the study hypotheses, OCD symptom severity (as measured by YBOCS), general anxiety symptoms (as measured by GAD-7), depressive symptoms (as measured by PHQ-9) and subjective distress during the entire MRI session (as measured by SUDS) were used as independent variables. These variables were person-mean and person centered in order to study between-person and within-person components of the clinical variables. Each of the clinical variables were modeled with the following dependent variables: behavioral variables were the SSRT and SucGoRT, while BOLD signal in the right IFG during successful stop-trials vs. successful go-trials was used as the dependent variable for task-related brain activity. Task-related functional connectivity was the psychophysiological interaction for connectivity between the right amygdala and the right pre-SMA during successful stop-trials vs. successful go-trials (see Figure 1 for an overview of the methods). As this was an exploratory study and the first of its kind to investigate variation in levels of brain activation and response inhibition after treatment, adjustment for multiple comparisons was not applied. This choice was made in order to facilitate novel discoveries, based on the recommendations of Bender and Lange (56) and Abramovitch and colleagues (18). They argue that exploratory studies should be performed with more lenient thresholds when there is little research to base confirmatory, theory-testing studies on. The reader should therefore bear in mind that the results should be interpreted cautiously, and need further investigation to be confirmed.

Data from three time points was available for 24 OCD patients, and five additional participants had data from two time points. As multilevel models do not require complete data sets (57), all the 29 OCD patients that had data for more than one time point were included in the analyses. There was one statistical outlier (SD > 3) on the SSRT variable at pre-treatment, but exclusion of this participant at that time point did not significantly alter the results of the analysis. Thus, in order to obtain as much statistical power as possible for each time point, the participant was included in the analysis.

## Results

### Clinical Changes Over TIME

After the B4DT, 18 (62.1%) of the patients were in remission, 7 (24.1%) were responders and 4 (13.8%) had no clinically significant change. Multilevel models showed that the patients' obsessive-compulsive symptoms showed a statistically significant decrease after treatment (*F* = 127.17, *p* < 0.01). Anxiety levels also showed a statistically significant decrease after treatment (*F* = 18.90, *p* < 0.01), and the same was true for depressive symptoms (*F* = 4.28, *p* = 0.02), but not for subjective distress during fMRI (*F* = 2.89, *p* = 0.06). See Table 2 for means and standard deviations at each time point.

**Table 2:** Means for clinical change and change in behavior, brain activation, and connectivity.

	**Mean (SD) pre-treatment**	**Mean (SD)3 days post-treatment**	**Mean (SD) 3 months post-treatment**	**Effect of time**	**Partial eta squared**
YBOCS	26.93 (4.15)	10.46 (5.95)	10.50 (6.41)	*F* (2, 57.27) = 127.17, *p* < .01	0.816
GAD-7	12.43 (5.18)	8.72 (4.63)	7.30 (4.74)	*F* (2 50.26) = 18.90, *p* < 0.01	0.429
PHQ-9	11.11 (5.95)	8.72 (5.89)	8.05 (5.32)	*F* (2, 48.44) = 4.28, *p* = 0.02	0.150
SUDS	27.25 (15.91)	23.65 (14.57)	20.51 (13.60)	*F* (2, 56.2) = 2.89, *p* = 0.06	0.093
SucGoRT	513.72 (106.02)	506.50 (116.72)	496.39 (102.35)	*F* (2, 55.44) = 0.72, *p* = 0.49	0.025
SSRT	197.31 (36.22)	193.45 (37.08)	191.88 (43.78)	*F* (2, 55.59) = 0.31, *p* = 0.73	0.011
R. IFG activation	1.12 (1.13)	0.95 (1.08)	0.97 (1.04)	*F* (2, 52.84) = 0.28, *p* = 0.76	0.010
R. amygdala – R. pre-SMA connectivity	0.37 (0.87)	−0.05 (1.02)	0.31 (1.23)	*F* (2, 82.00) = 1.36, *p* = 0.26	0.032

*GAD-7, Generalized Anxiety Disorder 7; IFG, Inferior frontal gyrus; PHQ-9, Patient Health Questionnaire-9; R, Right; SMA, supplementary motor area; SucGoRT, Response time on successful go trials; SSRT, Stop Signal Response Time; SUDS, Subjective Units of Distress Scale; YBOCS, Yale-Brown Obsessive Compulsive Scale*.

### SST Performance, Task-Related Activation, and Connectivity Over Time

Similar to Thorsen et al. (37), multilevel analyses showed that task performance, including SucGoRT (*F* = 0.72, *p* = 0.49) and SSRT (*F* = 0.31, *p* = 0.73) did not significantly change after treatment. There were also no significant changes for task-related activity in the right IFG (*F* = 0.28, *p* = 0.76) or connectivity between the right amygdala and the right pre-SMA (*F* = 1.36, *p* = 0.26). See Table 2 for means and standard deviations at each time point.

### Within-Person Effects

We found no significant within-person effects for task-related activation in the right IFG, or connectivity between the right amygdala and right pre-SMA.

### Between-Person Effects

Severity of obsessive-compulsive symptoms (B = 5.29, 95% CI [0.05–10.54], *p* = 0.048, R^2^ = 0.045), severity of anxiety (B = 5.78, 95% CI [0.63–10.94], *p* = 0.028, R^2^ = 0.080), and severity of depressive symptoms (B = 4.86, 95% CI [0.61–9.10], *p* = 0.026, R^2^ = 0.054) showed statistically significant positive correlations with SucGoRT (Table 3). Thus, when levels of obsessive-compulsive symptoms, anxiety and depression in patients are high across time, their SucGoRT in general is slower.

**Table 3:** Within- and between-person effects.

**Predictor variable**	**Outcome variable**	**Type of effect**	***B*** **(SE)**	***t***	***p***	**95% CI**		**R^**2**^**	**Covariance structure**	**Intercept & slope**
						**Lower**	**Upper**			
YBOCS	SucGoRT	BP	5.29 (2.64)	2.01	0.048	0.05	10.54	0.045	Diagonal	Fixed intercept, fixed slope
GAD-7	SucGoRT	BP	5.78 (2.59)	2.23	0.028	0.63	10.94	0.080	Diagonal	Fixed intercept, fixed slope
PHQ-9	SucGoRT	BP	4.86 (2.13)	2.28	0.026	0.61	9.10	0.054	Diagonal	Fixed intercept, fixed slope
SUDS	SucGoRT	*Not significant*								
YBOCS	SSRT	*Not significant*								
GAD-7	SSRT	*Not significant*								
PHQ-9	SSRT	*Not significant*								
SUDS	SSRT	*Not significant*								
YBOCS	R. IFG activation	*Not significant*								
GAD-7	R. IFG activation	*Not significant*								
PHQ-9	R. IFG activation	*Not significant*								
SUDS	R. IFG activation	*Not significant*								
YBOCS	R. amygdala – R. pre-SMA connectivity	*Not significant*								
GAD-7	R. amygdala – R. pre-SMA connectivity	*Not significant*								
PHQ-9	R. amygdala – R. pre-SMA connectivity	*Not significant*								
SUDS	R. amygdala – R. pre-SMA connectivity	*Not significant*								
R. amygdala – R. pre-SMA connectivity	SSRT	*Not significant*								
R. amygdala – R. pre-SMA connectivity	SucGoRT	*Not significant*								
R. IFG activation	SSRT	*Not significant*								
R. IFG activation	SucGoRT	*Not significant*								

*BP, Between-person effect; GAD-7, Generalized Anxiety Disorder 7; IFG, Inferior frontal gyrus; PHQ-9, Patient Health Questionnaire-9; R, Right; SMA, supplementary motor area; SucGoRT, Response time on successful go trials; SSRT, Stop Signal Response Time; SUDS, Subjective Units of Distress Scale; YBOCS, Yale-Brown Obsessive Compulsive Scale; WP, Within-person effect*.

## Discussion

The present study used a novel statistical framework to separate between- and within-person effects on task performance, task-related IFG activation and fronto-limbic connectivity in 29 OCD patients who performed a SST during fMRI 1 day before treatment, 3 days after treatment (1 week after the initial scan), and 3 months after treatment.

We found significant positive between-person relationships between obsessive-compulsive, anxiety, and depression symptom severity and SucGoRT, which persisted after symptom improvement. This indicates that patients with more severe obsessive-compulsive, anxiety, and depressive symptoms in general respond slower during successful go trials. These findings may point toward a stable relation between slower processing speed and generally higher levels of OCD symptoms.

OCD patients have been found to show mild to moderate problems in broad areas of executive function, including response inhibition and processing speed (7), and a recent meta-analysis showed that symptom severity to some extent may affect response time and other neuropsychological measures, though the degree of explained variance is likely small and the majority of studies did not find a significant association (58). There are inconsistent findings of treatment effects on neuropsychological performance, where some studies reported that pre-treatment abnormal task performance (compared with healthy controls) remained after CBT (25, 26, 31, 32), while other studies found improvements after CBT (27–30). One possible explanation for the inconsistent results in treatment studies may be that previous findings represent a mixture of between- and within-person effects. Our findings suggest that the higher severity in several symptom scales is related to longer SucGoRT, and this effect is independent of changes in symptom severity over time. However, the finding warrants replication in studies which separate within- and between person effects.

Few studies have investigated the relation between the severity level of obsessive-compulsive and anxiety symptoms, task performance, brain activation, and connectivity, leaving few comparable studies. As such, the findings warrant replication in an independent sample. There were no significant between- or within-person relationships between symptoms of depression or state anxiety and connectivity between the amygdala and the right pre-SMA. Thus, between-person variation in symptom severity does not seem to account for the connectivity differences between OCD patients and healthy controls found in other studies (23, 37).

There were also no significant within- or between-person relationships between any of the clinical variables and SSRT. Previous studies have pointed toward altered inhibitory performance as a possible trait in OCD (8–10). However, the null-finding of the present study is likely related to the fact that the patients in our sample did not show abnormal inhibitory performance compared with healthy controls (37). Considering that patients did not differ from controls before treatment, we find little evidence of poor inhibition as a trait in our sample. Furthermore, one would not expect a change after treatment when there was no pre-treatment difference between patients and controls. Thus, these findings warrant replication.

Even though previous studies (8, 37) found group differences between patients and healthy controls in inhibition-related BOLD response in the right IFG, the present study found no association between the mean level of severity of symptoms over time (between-person relationship) or intra-individual variation in levels of symptom scores (within-person relationship) and right IFG activation in the patient group. Thus, we found no evidence supporting that the previously observed group effects (8, 37) are mediated by general levels of symptom severity or that treatment-induced symptom reduction affects the level of IFG activation. This suggests that altered right IFG activation is a stable marker in OCD patients.

There are several limitations in this study. First, the sample size is small, warranting replication in larger samples. Second, the pre-treatment variation in YBOCS scores was low (all patients were moderately to severely affected), and the majority of the patients showed significant clinical improvement after the B4DT (with little variation in treatment response). Third, being an exploratory study, the results have not been corrected for multiple comparisons to avoid type II errors. The fourth limitation of this study is the number of measurement times. Using MLM, three time points is the minimum amount of measurements to perform analyses, and preferably one should include more time points than this. Our study design only included the minimum amount, which renders the results less robust than if we had more time points. This is especially true for the detection of within-person effects, and should be taken into consideration in the design of future studies that aim to replicate these findings.

Using disaggregation of within-person and between-person variables we have investigated potential longitudinal associations between general levels of, and variability in, clinical measures and response inhibition, related brain activity and functional connectivity. This type of analyses may offer further insight into whether and how general levels of obsessive-compulsive, anxiety, and depression symptom severity and subjective distress affect SST performance and related brain activation and connectivity, and whether the outcome variables are affected by intra-individual variability in the clinical predictors. This exploratory study found evidence for effects driven by between-person relationships between symptom severity and reaction time in OCD patients. Treatment studies should disaggregate between- and within-person effects of clinical variables to better understand how each of these affect inhibitory performance and related brain activation and connectivity over time in OCD.

## Data Availability Statement

The datasets for the study are not publicly available due to the restrictions by Norwegian legislation for privacy protection.

## Ethics Statement

The studies involving human participants were reviewed and approved by Regional Commitee for Medical and Health Research Ethics South-East. The patients/participants provided their written informed consent to participate in this study.

## Author Contributions

GK and BH developed the B4DT. GK, BH, OH, SW, and AT developed the fMRI study design. KH coordinated the study and did the clinical assessment. AT and PH carried out the data collection and performed data analyses. PH wrote the first draft of the manuscript. AT, OH, OO, RG, GK, BH, SW, and KH supervised, edited, and contributed to finalizing the manuscript. All authors contributed to the article and approved the submitted version.

## Conflict of Interest

OH has received speaker's honorarium by Benecke. The remaining authors declare that the research was conducted in the absence of any commercial or financial relationships that could be construed as a potential conflict of interest.
